# Cook County Hospital, Chicago, Ills.

**Published:** 1869-03-01

**Authors:** B. C. Miller

**Affiliations:** House Surgeon


### *Cook County Hospital, Chicago, Ill.*

Rupture of Urethra—Cystotomy—Cured—Service of Dr. Edwin Powell—B. C. Miller, House Surgeon.—Frank G., aged 60, native of Ireland, by occupation a grocer, admitted into the hospital October 9th, 1868. Habits irregular; previous health good. States that four weeks since he contracted gonorrhœa, five days after this was unable to micturate; he applied to a physician for relief and was catheterized with great difficulty. After this was unable to pass a drop of water, an instrument could not be passed. After suffering the most intense agony for some time he was suddenly relieved, and on examination his scrotum was terribly swollen. Four days after this, two large incisions were made into each side of the scrotum, liberating a large amount of water, pus and gangrenous shreds of tissue. When admitted to the hospital he was very much emaciated and depressed in body and mind. The scrotum presented a swollen, gangrenous appearance, and an odor so offensive that it was almost impossible for any one in the ward to lie in close proximity to him; on examination, found that the urine passed through the scrotum, none escaping through the urethra in front. In the perineum, extending as far back as the junction of the deep and superficial layer of the deep perineal fascia was a large fluctuating mass.

Oct. 12.—Patient restless during the night, cannot sleep, appetite poor, large sloughs came away, leaving the right testicle exposed. At least one-half of his scrotum gone.

Oct. 13.—Was placed under the influence of chloroform, and cystotomy preformed. (The operation was essentially the same as Dupuytren's lateral operation for stone.) On making the first incision through skin and superficial fascia a quantity of pus, urine and gangrenous cellular tissue was met by the operator. Not much hæmorrhage accompanied the operation. Patient reacted kindly although quite weak. He was given a full dose of morphine, and an ounce of brandy. Three hours after operation was quite comfortable.

Oct. 14.—Patient feeling quite comfortable; urine all passing through the incision. Removed another large slough from the scrotum, leaving both testicles exposed.

Oct. 16.—The sloughs all came away to-day, leaving a surface studded with healthy granulations, and the patient minus, at least calculation, two-thirds of his scrotum. Water all comes away through the incision. The patient able to hold the water for a time and pass it at will.

Oct. 19.—Is comfortable. Has complete control over the evacuation of bladder. Scrotum granulating finely.

Oct. 30.—Doing well. Good appetite and sleeps well.

Nov. 1.—Still improving. A No. five catheter passed into the bladder and allowed to remain, the water passing through it.

Nov. 5.—Removed the No. five catheter and introduced a a No. *ten*, and allowed it to remain. The wound caused by the operation granulating, is almost healed.

Nov. 9.—Wound completely healed. Scrotum doing well.

Nov. 26– Is gaining flesh rapidly. Scrotum healed to a small point, that communicating with the urethra.

Dec. 7.—Scrotum completely healed; catheter removed. All the water passing "*per vias naturales.*" The patient has been for years,the victim of a varicocele which caused him great inconvenience; on the scrotum healing and the patient regaining his feet, the varicocele was found to have disappeared. Whether the veins sloughed away with the scrotum, or whether the contraction caused by cicatrization of the organ cured them, I am unable to say.

Dec. 26.—Since admission to the hospital the patient has gained twenty-one and a quarter pounds. To-day he is discharged, cured.

